# EEG-based clustering shows distinct separation of chronic pain patients before spinal cord stimulation surgery

**DOI:** 10.1016/j.neuroimage.2026.122002

**Published:** 2026-05-13

**Authors:** Ayden Dunn, Jay Gopal, Marisa DiMarzio, Julie G. Pilitsis, Ilknur Telkes

**Affiliations:** a Charles E. Schmidt College of Medicine, Florida Atlantic University, Boca Raton, FL, USA; b The Warren Alpert Medical School of Brown University, Providence, RI, USA; c Department of Neurosurgery, University of Arizona College of Medicine - Tucson, Tucson, AZ, USA; d Department of Biomedical Engineering, University of Arizona, Tucson, AZ, USA

**Keywords:** Chronic pain, Electroencephalography, Clustering analysis, Preoperative assessment, Spinal cord stimulation

## Abstract

Chronic pain is associated with disrupted cortical activity, yet individual variability in these neural patterns remains poorly understood. Electroencephalography (EEG) provides a noninvasive means of characterizing these dynamics and may help identify patient subtypes relevant to spinal cord stimulation (SCS) outcomes. This study applied unsupervised machine learning to preoperative EEG to determine whether chronic pain patients exhibit distinct neurophysiological clusters and whether these groups differ in clinical characteristics or patient-reported outcomes (PROs). PROs included measures of pain intensity and related domains such as disability, catastrophizing, and mood, assessed using the Numerical Pain Rating Scale (NRS), Oswestry Disability Index (ODI), Pain Catastrophizing Scale (PCS), McGill Pain Questionnaire (MPQ), and Beck’s Depression Inventory (BDI). Resting-state scalp EEGs were recorded from 16 patients scheduled for SCS implantation. After standard preprocessing, spectral features were extracted and k-means clustering (K = 3) was applied to identify the structures within the EEG feature space. Three clusters emerged. Cluster 1 was characterized by globally reduced alpha and low-beta power along with lower theta power and entropy, suggesting a pattern of reduced oscillatory activity and spectral complexity within sensorimotor-related regions. In contrast, Cluster 2 showed elevated alpha and low-beta power, consistent with a distinct oscillatory profile that may reflect altered network dynamics. Cluster 3 exhibited moderate alpha and low-beta power alongside the highest theta entropy, indicating greater spectral complexity and variability in neural activity patterns, distinguishing this cluster from the other clusters and potentially relevant to pain processing. Demographic variables were similar across groups, but NRS “worst” scores differed significantly (p = 0.046). Feature-importance analysis identified peak low-beta and alpha power in primary somatosensory (S1), secondary somatosensory (S2), primary motor (M1), and parieto-occipital (PO) regions as the strongest contributors to cluster separation. Peak low-beta power across S1, S2, M1, and PO showed the most robust between-cluster differences (all p ≤ 0.004), with peak alpha power in S1 and M1 also differing significantly (p ≤ 0.008). Within clusters 2 and 3, multiple EEG features correlated significantly with postoperative improvements in PROs, suggesting potential neural markers of symptom change. Cluster-specific EEG features were associated with postoperative improvement, supporting the potential utility of EEG for identifying chronic pain phenotypes and informing individualized neuromodulation approaches.

## 1. Introduction

Spinal cord stimulation (SCS) has been established treatment option for refractory chronic pain, involving the implantation of electrodes in the epidural space and their associated stimulator placed in a subcutaneous pocket which can be controlled by an external remote to deliver pain relief (Caylor et al., 2019; Knotkova et al., 2021). This pain relief theoretically stems from activating inhibitory dorsal horn interneurons that reduce nociceptive transmission, along with modulation of supra-spinal pain networks (Knotkova et al., 2021; Sdrulla et al., 2018; Sun et al., 2021). Electroencephalography (EEG) can provide insights into the undercurrents of these networks and is critical for assessing neural dynamics of pain (Biasiucci et al., 2019). EEG was used by Bott et al. to show chronic pain intensity robustly associated with large-scale brain connectivity patterns spanning theta, alpha, and beta frequencies, particularly involving the limbic network (Bott et al., 2025). Dinh et al. further demonstrated that chronic pain patients exhibit lower theta power in the prefrontal cortex on EEG, and May et al. used EEG to find that chronic pain patients had reduced activity of the dorsolateral prefrontal and posterior parietal cortices (May et al., 2021; Ta Dinh et al., 2019). EEG has also been used in the context of SCS. For instance, Vanneste and De Ridder demonstrated reduced alpha and beta power in the dorsal anterior cingulate cortex and significantly reduced theta activity in the pregenual anterior cingulate cortex during burst stimulation in chronic pain patients (Vanneste and De Ridder, 2023).

Determining patient responsiveness to SCS involves various measures, commonly patient-reported outcome (PROs) such as the Numerical Pain Rating Scale (NRS), Oswestry Disability Index (ODI), Pain Catastrophizing Scale (PCS), McGill Pain Questionnaire (MPQ), and Beck’s Depression Inventory (BDI). There is substantial research in support of score changes within these PROs reflecting clinically significant reductions in chronic pain (Chapman et al., 2011; Chiarotto et al., 2018; Corallo et al., 2020; De Jaeger et al., 2021; ElSaban et al., 2023; Klasova et al., 2025; Sabourin et al., 2021). As chronic pain is a multi-dimensional condition, PROs are essential tools for identifying patterns of pain severity, disability, and psychological distress across individuals (Dansie and Turk, 2013). Yet, clinical self-report alone may not capture the full complexity of the underlying neurobiology. Therefore, variability observed across both PROs and EEG-derived neurophysiological measures necessitates robust analytic methods capable of identifying meaningful structure across clinical and neurophysiological domains.

Machine learning (ML) approaches have been increasingly applied to predict chronic pain outcomes, including pain reduction, disability, and quality of life. In particular, supervised ML models using EEG have demonstrated promising performance (62–100% accuracies) in classifying pain intensity, phenotypes, and treatment outcomes (Alazrai et al., 2019; Elsayed et al., 2020; Kimura et al., 2021; Nezam et al., 2018; Tripanpitak et al., 2020; Vijayakumar et al., 2017; Yu et al., 2020a, 2020b). In our prior work, supervised ML applied to intraoperative EEG achieved approximately 88% accuracy in predicting 3-month SCS pain relief (Gopal et al., 2025). However, supervised approaches rely on predefined outcome labels, which may be variable and influenced by subjective reporting. In contrast, unsupervised ML methods, such as cluster analysis, identify patterns innate to the dataset itself without requiring labeled outcomes (Everitt, 2011). These approaches have been applied in chronic pain to identify subgroups associated with differences in pain, disability, and quality of life (Alter et al., 2021; Gholi Zadeh Kharrat et al., 2025; Rijsdijk et al., 2025). Despite this, applications of unsupervised ML to EEG data in chronic pain remain limited (Levitt et al., 2020; Zolezzi et al., 2023). This gap is notable, as clustering can uncover hidden neural subtypes and reveal structure that does not align with conventional clinical classifications, without requiring large, labeled datasets.

Building on this gap, the objective of this study was to determine whether unsupervised clustering of preoperative scalp EEG recorded in sensor space could identify distinct neurophysiological subgroups of chronic pain patients undergoing SCS and whether these subgroups differ in clinical characteristics and PROs. Using EEG and survey data from 16 patients, we tested the hypothesis that EEG-derived clusters would exhibit distinct spectral profiles across sensorimotor and parieto-occipital regions and that these differences would be associated with variation in PROs.

## 2. Materials and methods

This study utilized de-identified records from 16 individuals with chronic pain who were scheduled for permanent SCS implantation at Albany Medical Center. The experimental protocol was approved by the Institutional Review Board of Albany Medical Center (IRB Number: 4973). Prior to surgery, data were gathered on demographic factors such as age and sex, clinical characteristics including primary diagnosis and duration of condition, and medication profiles documenting both prescribed dosages and calculated morphine milligram equivalents (MMEs). Baseline assessments also incorporated a comprehensive set of PROs, which included NRS, BDI, ODI, MPQ with sensory and affective components, and PCS with its corresponding subcomponents. PROs were additionally collected three months post-implantation to assess postoperative changes.

### 2.1. Acquisition of EEG and signal processing

Preoperative resting-state EEG was recorded from all participants using a 60-channel active electrode montage, with a reference at the earlobe and ground at AFz. Signals were sampled at 2400 Hz using FDA-cleared biosignal amplifiers (g.USBamp, g.tec medical engineering GmbH, Graz, Austria), with continuous streaming to a laptop for subsequent offline processing. Ten minutes of resting-state EEG was recorded while participants were seated in a comfortable upright position with back support, in a relaxed, eyes-closed state under dim ambient lighting. Electrode impedances were kept below 5 kOhm.

EEG and clinical data were connected through identifier codes that were randomly assigned and maintained consistently across all analysis stages. All analyses were conducted in sensor space using scalp EEG recordings. Each recording was visually evaluated for overall signal quality and to detect any artifacts. EEG preprocessing was conducted using a combination of EEGLAB functions, verified EEGLAB plugins, and custom MATLAB routines (Delorme and Makeig, 2004). Signals were band-pass filtered from 1–150 Hz using a 3rd-order Butterworth filter, with additional notch filters at 60 Hz and 120 Hz to suppress line noise and its harmonic (Tianxiao et al., 2017). Each recording was visually inspected to identify gross artifacts and assess overall signal quality. Independent component analysis (ICA) was applied using the extended Infomax algorithm implemented in EEGLAB. To automate artifact removal, we used the Multiple Artifact Rejection Algorithm (MARA) plugin (Winkler et al., 2011). Components associated with ocular, muscular, and cardiac artifacts were removed based on a probabilistic model. After ICA-based artifact rejection, data were re-referenced to the common average reference, a standard approach that reduces spatial bias and enhances signal comparability across electrodes (Gratton, 1998). Following artifact rejection, an average of 506.5 ± 138.1 s (~8.4 ± 2.3 min) of artifact-free EEG data per participant was retained for spectral analysis.

Frequency-domain features were extracted using a modified Welch periodogram approach. Power spectral density was estimated using a 1-second Hanning window and 50% overlap (Wilson et al., 2022). In contrast to the standard Welch method, which averages spectra across segments, we computed the median spectrum to reduce sensitivity to transient artifacts and outliers, consistent with our prior work (Gopal et al., 2025; Telkes et al., 2018). We calculated spectral entropy for the following frequency bands: theta (4–7 Hz), alpha (8–12 Hz), low-beta (15–22 Hz), beta (13–30 Hz), and gamma (30–70 Hz). To account for inter-individual variability due to non-neural factors such as skull thickness, electrode impedance, and volume conduction (Nunez et al., 2006), subband power was computed in logarithmic (dB) units relative to the high gamma band (100–150 Hz), defined as:

$${\text{P}}_{\text{rel}}\left(\text{f}\right)=10*{\text{log}}_{10}\left(\text{P}\left(\text{f}\right)/{\text{P}}_{100-150\text{Hz}}\right),$$

where *P*(*f*) denotes the power within a given frequency band and *P*_100−150*Hz*_ represents the average power within the high-gamma reference band. This frequency range was selected as a broadband reference band that is less dominated by canonical oscillatory rhythms (e.g., alpha, beta) and instead reflects more aperiodic, broadband components of the EEG signal (Buzsaki and Wang, 2012; Miller et al., 2007). Furthermore, this approach provides a broadband normalization baseline while avoiding overlap with the analyzed gamma band (30–70 Hz) and improves comparability across subjects for downstream clustering analysis.

Peak power (defined as the maximum amplitude within each subband), peak frequency (defined as the frequency of maximal power), and alpha-to-theta (AT) peak power ratio were additionally computed. These features were selected to capture complementary aspects of oscillatory dynamics relevant to chronic pain neurophysiology, including band-specific power, dominant frequency shifts, and cross-band relationships. Alterations in oscillatory power (particularly in the alpha and theta ranges) and a shift toward lower peak frequencies have been associated with disrupted thalamocortical dynamics in chronic pain (de Vries et al., 2013; Sarnthein et al., 2006), while peak alpha frequency has been shown to correlate with individual differences in pain sensitivity (Furman et al., 2018). Cross-band relationships such as the AT ratio were utilized to capture cross-band dynamics that may not be fully reflected by absolute power alone. These ratios reflect the relative distribution of activity across frequency bands and have been associated with pain severity and the cognitive-affective processing of pain (Ploner et al., 2017), consistent with our prior work (Telkes et al., 2020a) (Gopal et al., 2025).

Dimensionality reduction was applied to address high dimensionality of the feature space relative to sample size by focusing on the theta, alpha, and low-beta bands, as well as the AT peak power ratio and five anatomically defined regions of interest (ROIs), namely the prefrontal cortex (PFC), the primary motor cortex (M1), the primary somatosensory cortex (S1), the secondary somatosensory cortex (S2), and the parieto-occipital cortex (OP) regions, based on prior work (Gopal et al., 2025).

### 2.2. Clustering analysis

**Fig. 1.**
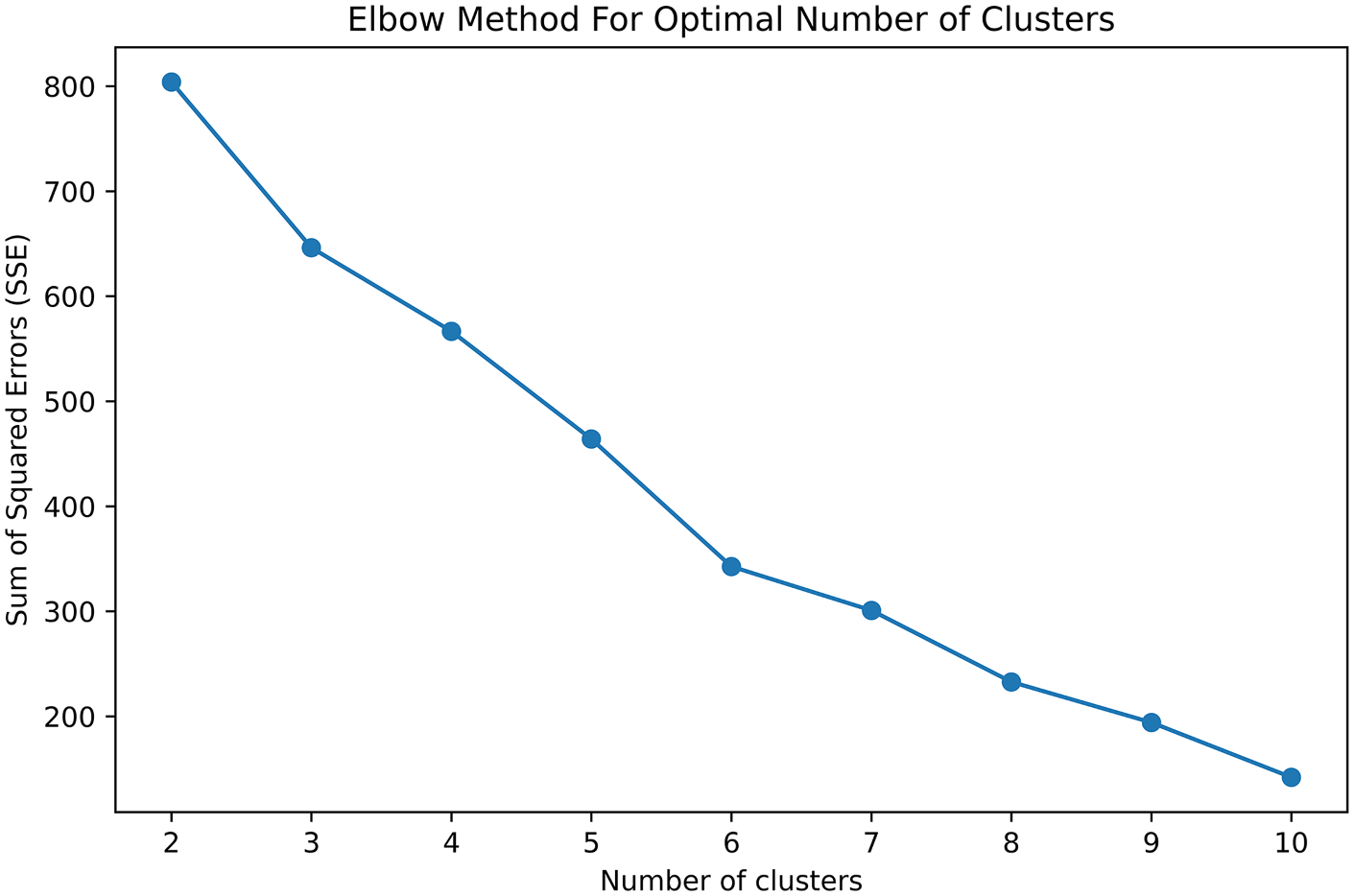
Elbow method used to identify the optimal number of clusters for K-means clustering. The curve depicts the within-cluster sum of squares as a function of k.

EEG features were analyzed using an unsupervised ML pipeline to identify latent patient subgroups with distinct neurophysiological profiles. To reduce dimensionality and mitigate feature redundancy, Principal Component Analysis (PCA) was applied to the standardized EEG feature matrix. The optimal number of components was determined using the elbow method, which identified a plateau in explained variance. The first two principal components (PC1 and PC2) were visualized to assess separability among data points, and feature loading vectors were plotted to examine the contribution of individual EEG metrics to variance structure. K-means clustering was performed on the PCA-reduced feature set to identify distinct patient subgroups. The optimal cluster number (K = 3) was selected based on the within-cluster sum of squares and silhouette analysis, alongside qualitative elbow method analysis shown in Fig. 1 (Brusco, 2006; Lengyel and Botta-Dukat, 2019). All clusters identified by the unsupervised algorithm were retained for analysis to preserve the data-driven nature of the clustering approach.

Clusters were subsequently compared on demographic and clinical variables, including PROs. Demographic characteristics and PROs among clusters were assessed using both Kruskal-Wallis testing and qualitative analysis. Means and standard deviations were calculated for all demographic features and PROs.

Feature importance was computed using the average Euclidean distance of feature values between cluster centroids, allowing identification of EEG metrics most responsible for cluster separation. The top ten features by importance were further evaluated for statistical significance across clusters, first using the Kruskal-Wallis test to assess overall group differences, followed by Tukey-Kramer post hoc analysis for pairwise cluster comparisons. Topographical EEG maps were generated to visualize spatial patterns of the most discriminative bands. Finally, correlation analyses using the MATLAB *corrcoef* function were performed between cluster-specific EEG features and improvements in PROs from baseline to 3-month follow-up to explore potential neural predictors of treatment responsiveness. All correlations that reached significance were tabulated and reported.

All statistical analyses and data visualizations were performed using MATLAB R2024b (MathWorks, Natick, MA). All statistical tests were two-tailed, and significance was determined at an alpha level of 0.05. Given the exploratory nature of this study, formal correction for multiple comparisons was not applied.

## 3. Results

**Fig. 2.**
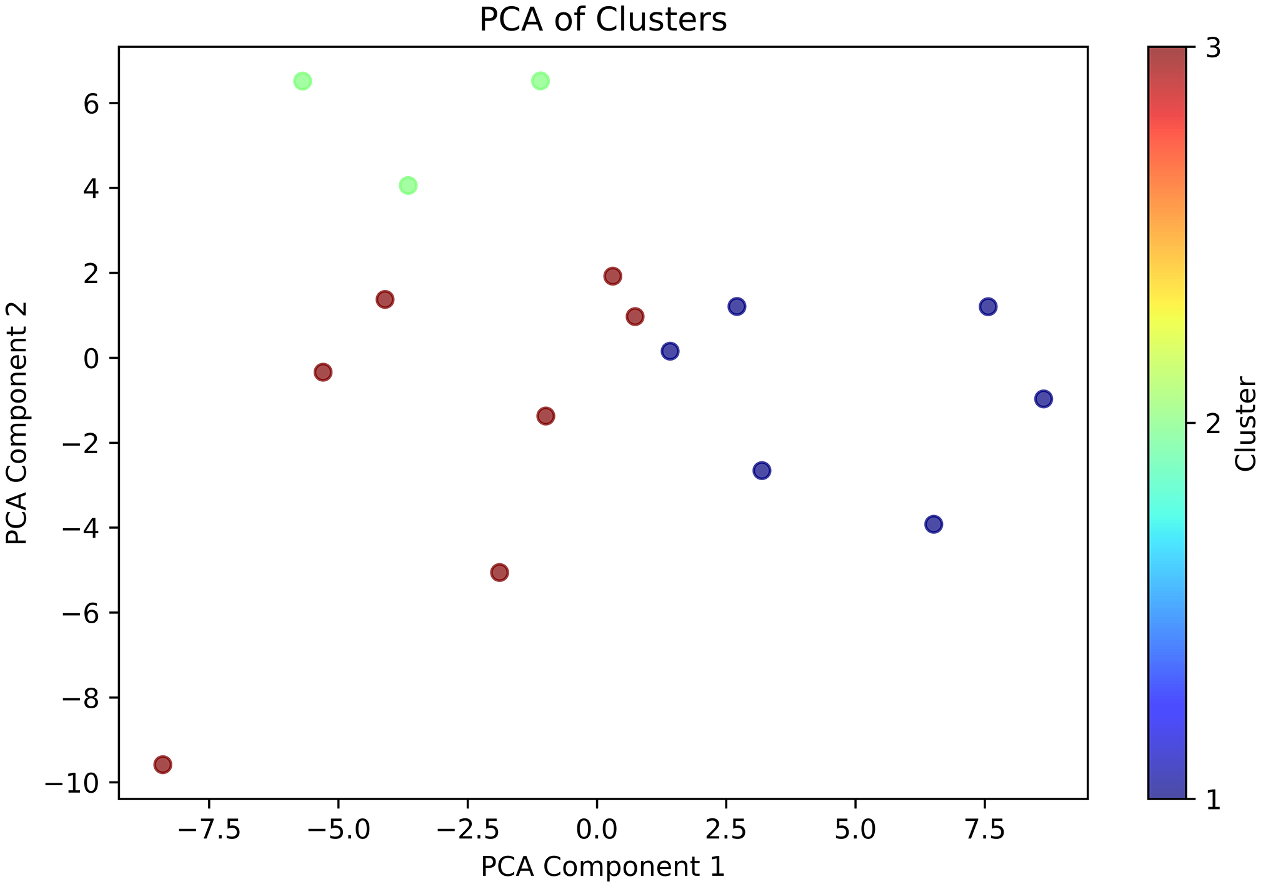
PCA visualization of EEG-based clusters. Scatter plot of top two principal components (PC1 vs PC2) showing the distribution of patients across clusters. Each point represents an individual, and colors indicate cluster membership derived from K-means clustering.

Application of the elbow method to the within-cluster sum of squared errors revealed a notable elbow point at K = 3, indicating that three clusters provided an optimal balance between model complexity and cluster cohesion (Fig. 1). This informed the selection of the number of clusters for subsequent K-means analysis. PCA was then used to visualize the structure of the high-dimensional EEG feature space. The first two PCs captured the majority of variance attributable to differences in spectral patterns, and the resulting scatter plot demonstrated clear spatial separation between clusters (Fig. 2). The visualization supported the presence of three distinct neurophysiological subgroups, with minimal overlap among clusters in the PCA-reduced space.

### 3.1. Participant characteristics across clusters

Our study included a total of 16 participants, comprised of 5 males and 11 females. Ages of participants ranged from 43 to 83 with a mean age of 65.27 years old. Participants were primarily diagnosed with one of two chronic pain etiologies: spinal pain syndrome (PSPS) type I (n = 10), including chronic neuropathic pain and lumbar radiculopathy without prior spine surgery, or PSPS type II (n = 6), including failed back surgery syndrome. The duration of each participant’s disease at study enrollment varied widely with a range of 4 to 40 years and a mean of 16.01 years. Participants reported a wide range of preoperative opioid use, measured in MME. Four participants reported no prescription opioid use, while the remaining 12 had MME values ranging from 5 to 82.5, with a mean of 23.41.

A summary of demographic and clinical characteristics by cluster (K = 3) is presented in Table 1. Although descriptive differences were observed across clusters, mean age was comparable among Cluster 1 (n = 2), Cluster 2 (n = 6), and Cluster 3 (n = 8) (67.50 ± 13.44, 64.67 ± 9.09, and 63.63 ± 14.32 years, respectively; *p* = 0.909). Disease duration (12.13 ± 0.18, 18.15 ± 11.99, and 15.38 ± 6.88 years, respectively; *p* = 0.445) and preoperative MME (13.75 ± 12.37, 29.08 ± 28.31, and 21.56 ± 25.74, respectively; *p* = 0.644) also varied descriptively but did not differ significantly across clusters based on Kruskal-Wallis testing. Thus, while descriptive differences were apparent, demographic and clinical factors alone do not explain the cluster structure, suggesting that the EEG features capture neurophysiological variation not reflected in standard clinical measures.

Additional trends in PROs across clusters are summarized in Table 2. While most measures did not reach statistical significance, pain intensity scores in the NRS “worst” subscale differed significantly between clusters (*p* = 0.046), with Cluster 1 reporting the highest average pain (9.50 ± 0.71), followed by Cluster 2 (8.83 ± 0.98) and Cluster 3 (7.81 ± 0.84). Other pain measures, including NRS “average” and NRS “now” followed a similar pattern, with Cluster 1 tending to report higher scores than Cluster 2 or 3, though these differences were not statistically significant. Measures of depression (BDI), disability (ODI), and pain catastrophizing (PCS) did not significantly differ between clusters, though modest variations in mean values were observed. For example, ODI scores were comparable across clusters, ranging from 46.50 ± 12.97 to 51.83 ± 18.83, and BDI scores showed a non-significant trend toward lower depression severity in Cluster 3 (12.88 ± 7.20) compared with Clusters 1 (19.00 ± 16.97) and 2 (18.83 ± 15.03). Overall, these findings suggest that while EEG-based clustering captures meaningful differences in pain intensity, broader psychosocial measures appear more uniformly distributed across clusters. While variations in pain scores were observed across clusters, no single clinical variable fully accounted for cluster membership, suggesting that EEG-derived groupings may capture neurophysiological variability not readily reflected in conventional clinical measures. Visual inspection of individual-level data (Supplementary Table S1) did not suggest that the identified clusters were driven solely by a small number of extreme cases, although this observation should be interpreted cautiously given the small sample size.

### 3.2. EEG patterns across clusters

Examination of the EEG features using the importance metric yielded several notable findings. Table 3 summarizes the ten features with the highest importance values. The six highest-ranked features consisted primarily of the peak power in low-beta and alpha bands, with ROIs localized to S1, S2, M1, and OP. The rest of the top-ranked features were derived from the theta band only, including entropy in S1, S2, and OP and peak frequency in OP region.

Kruskal-Wallis tests indicated significant differences among clusters for the seven top-ranked EEG features. Post-hoc Tukey-Kramer analysis revealed significant differences between Cluster 1 and Cluster 2 and between Cluster 2 and Cluster 3 across all low-beta features, suggesting that Cluster 2 exhibited a distinct low-beta profile. For alpha band features, Tukey-Kramer post-hoc testing identified significant differences between Clusters 1 and 2; however, no significant differences were found between Clusters 2 and 3. In addition, theta band spectral entropy differed significantly between Clusters 1 and 3. Taken together, these results indicate that each cluster is characterized by distinct EEG spectral properties spanning multiple frequency bands.

**Fig. 3.**
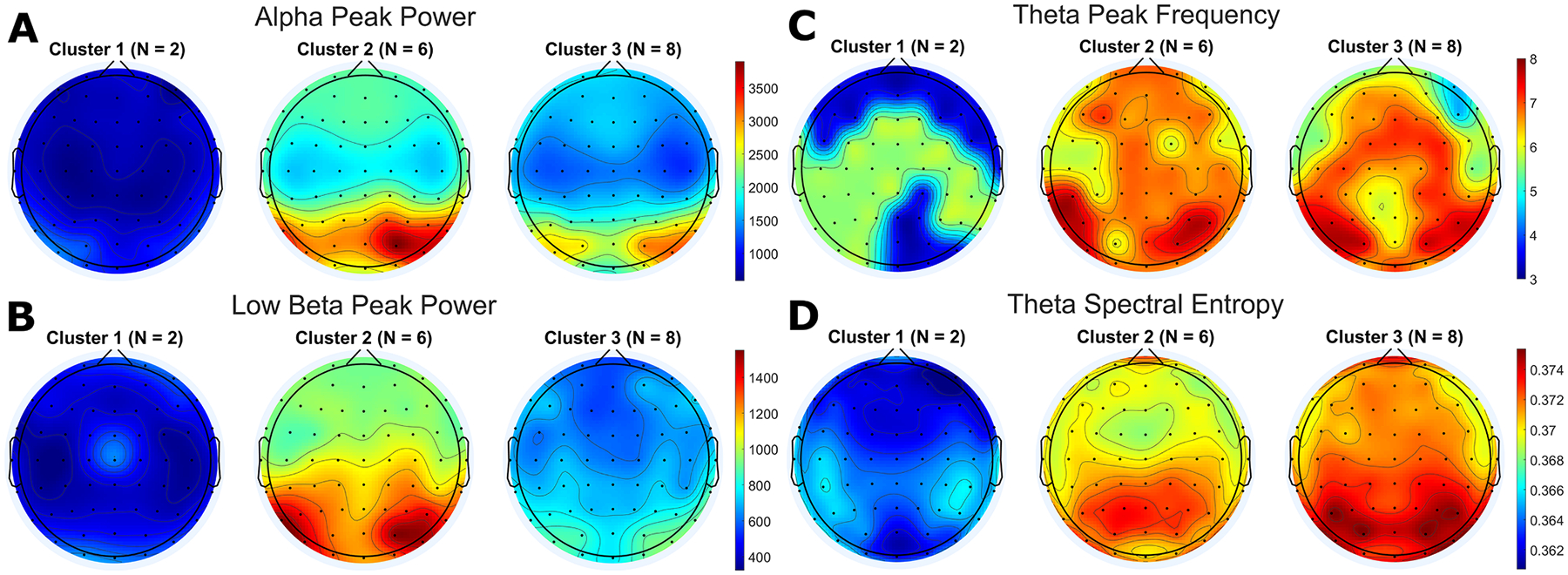
Topographical distributions of top spectral features across clusters. Scalp topography maps depict the spatial distribution of alpha peak power **(A)**, low-beta peak power **(B)**, theta peak frequency **(C)**, and theta band spectral entropy **(D)** in each cluster. Each map represents the cluster-averaged feature values, highlighting differences in cortical activity across frequency bands and cluster groups.

Topographical maps of cluster-averaged EEG features are shown in Fig. 3. Peak alpha power revealed clear differences across clusters (Fig. 3A). Cluster 1 exhibited uniformly low alpha peak power across all regions. In contrast, Cluster 2 demonstrated the highest alpha peak power, with prominent increases in OP and extensions into central areas. Cluster 3 showed alpha peak power values greater than those observed in Cluster 1, but consistently lower than Cluster 2, with a similarly posterior-dominant spatial distribution. Overall, alpha peak power displayed a graded pattern across clusters, suggesting that posterior alpha activity contributed to cluster separation.

Low-beta peak power topographies showed marked cluster-specific differences (Fig. 3B). Cluster 1 was again characterized by globally reduced low-beta peak power. Cluster 2 exhibited substantially elevated low-beta peak power across the scalp regions, with pronounced increases in posterior regions. Cluster 3 demonstrated moderate low-beta peak power, with values consistently lower than Cluster 2 but higher than Cluster 1. These spatial patterns indicate that low-beta activity strongly differentiated Cluster 2 from the other clusters, consistent with statistical analyses identifying low-beta features as key contributors to cluster separation.

Topographical maps of theta band peak frequency revealed clear differences across clusters (Fig. 3C). Cluster 1 exhibited generally lower theta peak frequencies across the scalp, with limited spatial variability. In contrast, both Cluster 2 and Cluster 3 showed higher theta peak frequencies, with broadly elevated values across posterior and central regions. The spatial distributions of theta peak frequency in Clusters 2 and 3 appeared qualitatively similar, suggesting that theta peak frequency primarily distinguished Cluster 1 from the other two clusters rather than separating Clusters 2 and 3 from each other.

Finally, theta band spectral entropy demonstrated pronounced cluster-specific patterns (Fig. 3D). Cluster 1 showed uniformly low entropy values across all regions, indicating more regular or less complex theta activity. Cluster 2 exhibited intermediate entropy levels, with regional increases most evident in posterior regions. In contrast, Cluster 3 displayed the highest theta spectral entropy, particularly in the OP region, which may reflect greater signal complexity. These patterns indicate that theta spectral entropy differentiated Cluster 3 from both Clusters 1 and 2 and contributed to the overall separation between clusters.

In summary, the spatial patterns observed in Fig. 3 are consistent with the feature-importance analysis in Table 3, which identifies low-beta peak power as the primary driver of cluster separation, with alpha band features contributing selectively and theta band entropy providing complementary differentiation.

### 3.3. Within-cluster associations between EEG features and PROs

Correlation analyses between EEG features and improvement in PROs from baseline to 3-month follow-up were performed only for Clusters 2 and 3 as Cluster 1 contained only 2 patients. Multiple EEG-PRO feature pairs demonstrated significant correlations within Cluster 2 (Table 4) and Cluster 3 (Table 5). Across both clusters, several ROIs showed significant negative correlations with improvement in the NRS “least” subscale.

In Cluster 2, negative correlations with NRS “least” improvement were observed for relative low-beta power in M1 (*r* = −0.929, *p* = 0.007); peak low-beta power in S2 (*r* = −0.885, *p* = 0.019), PFC (*r* = −0.837, *p* = 0.038), and the OP (*r* = −0.815, *p* = 0.048); AT peak power ratio in OP (*r* = −0.818, *p* = 0.047); and theta band spectral entropy in OP (*r* = −0.878, *p* = 0.021). Cluster 3 exhibited additional significant negative correlations with NRS “least” improvement, including peak theta power in S1 (*r* = −0.717, *p* = 0.045) and S2 (*r* = −0.823*, p* = 0.012), as well as peak low-beta power in S1 (*r* = −0.718, *p* = 0.045).

Cluster 2 also demonstrated significant associations with improvements in PCS and subscales. Improvements in PCS-total were negatively correlated with peak low-beta frequency in S2 (*r* = −0.831, *p* = 0.041), with similar negative correlations observed for PCS-rumination (*r* = −0.836, *p* = 0.038) and PCS-magnification (*r* = −0.853, *p* = 0.031). In addition, improvements in the MPQ-sensory subscale were positively correlated with low-beta spectral entropy in OP (*r* = 0.819, *p* = 0.047). In Cluster 3, significant positive correlations were observed between improvements in the NRS “worst” subscale and the AT peak power ratio in M1 (*r* = 0.752, *p* = 0.031), as well as between improvements in BDI scores and theta band spectral entropy in M1 (*r* = 0.707, *p* = 0.049).

## 4. Discussion

In this study, we demonstrate that preoperative resting-state EEG can uncover distinct, data-driven subgroups of chronic pain patients undergoing SCS. Using unsupervised clustering of a high-dimensional spectral feature set, we identified three neurophysiologically distinct patient groups, characterized by differences in alpha, low-beta, and theta activity across somatosensory, motor, and parieto-occipital regions. Notably, these clusters were not explained by demographic or clinical variables alone, suggesting that they reflect neurophysiological variability rather than clinically defined subgroups, and showed selective differences in pain intensity measures along with cluster-specific associations between EEG features and postoperative improvements in pain and psychological outcomes. These differences were primarily driven by alpha and low-beta activity across sensorimotor and parieto-occipital regions, consistent with prior work implicating altered cortical oscillatory dynamics in chronic pain. Together, these findings suggest that resting-state EEG may capture latent neurophysiological variation relevant to chronic pain that is not reflected in conventional clinical assessment and may provide a foundation for developing physiology-informed approaches to patient characterization as a potential step towards more personalized SCS treatment strategies.

### 4.1. Spectral feature selection

The spectral features selected for clustering were derived from well-established associations between neural oscillations and pain processing. Prior work has consistently linked alpha, theta, and beta rhythms to nociceptive signaling, pain modulation, and cognitive-affective aspects of pain perception (Bott et al., 2025; Mussigmann et al., 2022; Nickel et al., 2017, 2022; Pinheiro et al., 2016; Zebhauser et al., 2023). Alterations in these frequency bands, including reduced prefrontal theta power, altered somatosensory alpha activity, and changes in beta band synchrony, have each been reported across chronic pain cohorts, reflecting disrupted neural dynamics associated with persistent pain states (Fauchon et al., 2022; Makowka et al., 2023; Malan et al., 2025; Mussigmann et al., 2022; Simis et al., 2022). These same spectral features have also shown relevance to SCS, with prior studies reporting increases in alpha power in responders, modulation of beta band activity, and normalization of network connectivity within pain-relevant cortical regions (e.g., somatosensory cortices, dorsal anterior cingulate cortex) (Deogaonkar et al., 2016; Goudman et al., 2020; Telkes et al., 2020b; Vanneste and De Ridder, 2023; Witjes et al., 2023). Accordingly, our focus on spectral power within somatosensory, motor, parietal, and frontal regions was grounded by converging evidence that these frequency bands and ROIs encode clinically relevant dimensions of pain experience and may reflect meaningful variation relevant to SCS mechanisms.

### 4.2. EEG clustering in the context of clinical data

In this study, EEG-based clustering did not align with conventional clinical data, suggesting that EEG captures aspects of pain that are not readily observable through PROs or standard clinical descriptors alone. This dissociation aligns with prior work showing that subjective pain ratings often map weakly onto objective neural markers (Hoeppli et al., 2022; Mouraux and Iannetti, 2018; Robinson et al., 2013). Rather than reflecting a limitation of either modality, this divergence could point to complementary information being contained within both neurophysiologic (e.g., EEG, fMRI) and subjective measures. Thus, our EEG-based clustering may be uncovering neural differences among chronic pain patients that are not detectable using conventional clinical descriptors alone.

A 2020 study by Levitt et al. provides an additional important touchstone for interpreting our clustering results (Levitt et al., 2020). In that study, authors showed that conventional EEG metrics often fail to survive strict multiple-comparison correction, however more nuanced ML classifiers can reveal discriminative information about pain states, such as the low-gamma transient events localized to sensorimotor regions. This may further support the notion that EEG data contains subtler signatures of pain or neural patterns not otherwise apparent from PROs or summary statistics alone. At the same time, Levitt et al. emphasize that meaningful clinical translation requires larger, multi-site validation cohorts, transparent and interpretable feature representation, and integration of network-level metrics with behavioral and clinical measures to form a holistic and robust composite measure of chronic pain/treatment outcomes. Our findings align with this perspective, reinforcing the view that EEG-based clustering should be considered a complementary, exploratory approach rather than a standalone clinical classifier.

### 4.3. Cluster analysis and unsupervised learning

Unsupervised clustering offers a complementary perspective to supervised models that have been used to predict SCS outcomes. In previous work, we combined intraoperative EEG with feature selection to achieve high accuracy in predicting pain relief; however, these supervised models required labeling patients as responders or nonresponders based on subjective postoperative outcomes (Chen et al., 2024; Gopal et al., 2025). Such labels can vary over time and may be influenced by psychological or contextual factors, including variability in pain reporting, day-to-day symptom fluctuations and expectancy effects (Schneider et al., 2012; Vachon-Presseau et al., 2018). In contrast, unsupervised clustering allows the data to define its own structure without relying on predefined outcome categories. This approach is particularly relevant in chronic pain, where the underlying neural processes can diverge substantially even when two patients report similar levels of pain or disability (Mussigmann et al., 2022). By removing dependence on predetermined labels, clustering may help uncover neurophysiological patterns that are not captured by conventional clinical groupings and may offer complementary insights into underlying cortical network organization. The fact that our EEG-based clusters differed in NRS “worst” pain but did not show strong separation across broader psychosocial measures, suggests that the extracted neural patterns may reflect dimensions of pain-related physiology that are not fully captured by traditional PROs.

The cluster-specific EEG-PRO associations we observed here provide additional insight into the biological meaning of these subgroups. The distinct correlation patterns within Clusters 2 and 3 suggest that relationships between preoperative neural activity and postoperative outcomes are not uniform across patients even when they fall within the same overall SCS candidate population. For example, the negative associations between low-beta power and pain improvement in Cluster 2 may suggest that elevated sensorimotor region beta activity is associated with reduced benefit from SCS, whereas associations involving AT ratio or theta entropy in Cluster 3 point to a different set of neural constraints shaping responsiveness (Mussigmann et al., 2022). These subgroup-specific relationships may be attenuated or obscured in pooled supervised models, which assume a single mapping between EEG features and clinical outcomes. Instead, our clustering approach highlights the possibility that SCS may engage multiple physiological pathways across patients, with each pathway tied to distinct spectral signatures. While exploratory, these results suggest that EEG-based phenotyping may provide a framework for future efforts in developing individualized neuromodulation strategies, where stimulation settings, electrode configurations, or waveform selections are optimized according to the neural characteristics of a given subgroup rather than the average properties of a broad patient cohort.

### 4.4. Limitations and future directions

This study has several limitations that should be considered when interpreting the findings. First, the sample size was modest, limiting statistical power and constraining the process of cluster assignment. This aspect is particularly limiting for detecting smaller effect sizes or more nuanced subgroups. Additionally, with a smaller sample size, identified clusters may be unstable, dataset-specific, or could reflect incidental structure rather than reproducible subgroups. In such settings, clustering solutions may be sensitive to sampling variability and prone to overfitting, potentially capturing spurious structure rather than stable underlying subtypes. Although unsupervised clustering can reveal latent structure even in small datasets, replication in larger, multi-site cohorts will be essential to validate the reproducibility of the three-cluster solution reported here.

Second, our clustering relied exclusively on resting-state EEG features, which capture broad oscillatory differences but do not reflect transient spectral events, dynamic network connectivity, or cross-frequency interactions, which are features known to encode important aspects of pain processing (Kim and Davis, 2021; Kucyi and Davis, 2017; Liu et al., 2015). Incorporating connectivity and event-based metrics in future studies may yield richer neural profiles and improve subtype resolution (Bentley et al., 2016; Gamal-Eltrabily et al., 2021; Gopal et al., 2025; Tran et al., 2024; Wang et al., 2021).

Additionally, multiple statistical comparisons were performed without formal correction, which increases the risk of type I errors, and analyses were conducted in sensor space without source localization. As such, findings should be considered exploratory and regional interpretations should be viewed as approximate rather than anatomically precise.

Finally, while this study was conducted within a hypothesis-driven framework informed by prior work (Gopal et al., 2025; Hadanny et al., 2022; Telkes et al., 2020a) and funded project objectives (NIH R00NS119672), the analysis was not prospectively registered nor governed by a formally pre-specified analysis protocol. Although feature selection and analytical approaches were guided by existing literature and preliminary data, the clustering results were data-driven and did not align with our initial expectation of separating clinically defined groups (e.g., responders vs non-responders). Accordingly, the findings should be interpreted as exploratory and hypothesis-generating. Future studies with larger cohorts and prospectively defined analysis plans will be important to validate the reproducibility and clinical relevance of these observations.

## 5. Conclusion

In summary, this study suggests that unsupervised clustering of preoperative resting-state EEG can reveal neurophysiologically distinct subgroups of chronic pain patients undergoing SCS, characterized primarily by differences in alpha, theta, and low-beta activity. Although these EEG-defined clusters did not map onto demographic or clinical profiles, they showed preliminary associations with postoperative improvements in pain and psychological outcomes, suggesting that resting-state EEG may capture aspects of chronic pain heterogeneity not reflected by PROs alone. These findings highlight the potential utility of EEG-based phenotyping and unsupervised learning as exploratory tools for characterizing neural variability in chronic pain. By establishing a data-driven framework for identifying neurophysiological structures prior to implantation, this work offers a foundation for future studies aiming to integrate neural biomarkers into SCS research and evaluation, including investigation of patient heterogeneity, outcome variability, and treatment mechanism.

## Supplementary Material

1

Supplementary material associated with this article can be found, in the online version, at doi:10.1016/j.neuroimage.2026.122002.

## Figures and Tables

**Table 1:** Demographics and clinical characteristics of the clusters.

	Cluster 1 (n = 2)	Cluster 2 (n = 6)	Cluster 3 (n = 8)	P-value
Sex (n)				
Male:Female	2:0	2:4	1:7	
Age (years)	67.50 ± 13.44	64.67 ± 9.09	63.63 ± 14.32	0.909
Diagnosis (n)				
PSPS Type I	0	4	6	
PSPS Type II	2	2	2	
Duration of Disease (years)	12.13 ± 0.18	18.15 ± 11.99	15.38 ± 6.88	0.445
Baseline MME	13.75 ± 12.37	29.08 ± 28.31	21.56 ± 25.74	0.644

PSPS, Persistent Spinal Pain Syndrome; MME, morphine milligram equivalents. Categorical data are presented as counts, continuous data are presented as means ± standard deviation. P-values were derived via Kruskal Wallis testing. None of the analyses reached statistical significance (α = 0.05).

**Table 2:** Patient-reported outcome measures within clusters.

	Cluster 1 (n = 2)	Cluster 2 (n = 6)	Cluster 3 (n = 8)	P-value
NRS – worst	9.50 ± 0.71	8.83 ± 0.98	7.81 ± 0.84	**0.046**
NRS – least	5.50 ± 4.95	5.00 ± 2.76	4.06 ± 2.37	0.689
NRS – average	8.00 ± 0.00	7.58 ± 1.69	6.00 ± 1.83	0.059
NRS – now	7.00 ± 2.83	6.50 ± 3.67	5.44 ± 2.82	0.569
BDI	19.00 ± 16.97	18.83 ± 15.03	12.88 ± 7.20	0.732
MPQ – total	6.50 ± 2.12	4.67 ± 2.73	6.38 ± 0.92	0.480
MPQ – sensory	6.00 ± 1.41	4.33 ± 2.50	4.88 ± 2.03	0.622
MPQ – affective	0.50 ± 0.71	0.33 ± 0.52	1.50 ± 1.07	0.066
PCS – total	21.50 ± 30.41	22.00 ± 13.30	23.00 ± 12.43	0.982
PCS – rumination	7.50 ± 10.61	8.17 ± 3.97	9.38 ± 5.10	0.915
PCS – helplessness	9.50 ± 13.44	10.17 ± 8.26	10.00 ± 6.46	0.946
PCS – magnification	4.50 ± 6.36	3.67 ± 2.16	3.62 ± 2.62	0.993
ODI	50.00 ± 31.11	51.83 ± 18.83	46.50 ± 12.97	0.756

NRS, Numerical Pain Rating Scale; ODI, Oswestry Disability Index; PCS, Pain Catastrophizing Scale; MPQ, McGill Pain Questionnaire; BDI, Beck’s Depression Inventory. PROs are presented as means ± standard deviations. P-values were derived via Kruskal-Wallis testing. Significant p-values (α = 0.05) are shown in bold.

**Table 3:** Ten highest-ranked features ranked by importance.

Features	Importance	P-value	Significant Differences between Clusters
Peak low-beta power in S2	2.977	**0.002**	1 and 2, 2 and 3
Peak low-beta power in S1	2.872	**0.002**	1 and 2, 2 and 3
Peak alpha power in S1	2.816	**0.006**	1 and 2
Peak alpha power in M1	2.787	**0.008**	1 and 2
Peak low-beta power in M1	2.709	**0.002**	1 and 2, 2 and 3
Peak low-beta power in OP	2.630	**0.004**	1 and 2, 2 and 3
Theta entropy in OP	2.590	**0.044**	1 and 3
Theta entropy in S1	2.529	0.076	None
Peak theta frequency in OP	2.441	0.154	None
Theta entropy in S2	2.429	0.082	None

S1, primary somatosensory cortex; S2, secondary somatosensory cortex; M1, primary motor cortex; OP, parieto-occipital cortex. Kruskal-Wallis testing was used to determine p-values followed Tukey-Kramer post-hoc analysis. Significant differences (α = 0.05) are shown in bold.

**Table 4:** Significant correlations within cluster 2.

PROs	EEG Features	R-value	P-value
NRS – least	Relative low-beta power in M1	−0.9290	**0.007**
	Peak low-beta power in S2	−0.8847	**0.019**
	Peak low-beta power in PFC	−0.8372	**0.038**
	Peak low-beta power in OP	−0.8146	**0.048**
	AT Peak Power Ratio in OP	−0.8179	**0.048**
	Theta entropy in OP	−0.8785	**0.021**
MPQ – sensory	Low-beta entropy in OP	0.8189	**0.047**
PCS – total	Peak low-beta frequency in S2	−0.8307	**0.041**
PCS – rumination	Peak low-beta frequency in S2	−0.8362	**0.038**
PCS – magnification	Peak low-beta frequency in S2	−0.8534	**0.031**

NRS, Numerical Pain Rating Scale; PCS, Pain Catastrophizing Scale; MPQ, McGill Pain Questionnaire; AT, alpha-to-theta; S2, secondary somatosensory cortex; M1, primary motor cortex; OP, parieto-occipital cortex; PFC, prefrontal cortex. Significant correlations (α = 0.05) are shown in bold.

**Table 5:** Significant correlations within cluster 3.

PROs	EEG Feature	R-value	P-value
NRS – least	Theta peak power in S1	−0.7172	**0.045**
	Theta peak power in S2	−0.8234	**0.012**
	Low-beta peak power in S1	−0.7178	**0.045**
NRS – worst	AT peak power ratio in M1	0.7523	**0.031**
BDI	Theta entropy in M1	0.7074	**0.049**

NRS, Numerical Pain Rating Scale; BDI, Beck’s Depression Inventory; AT, alpha-to-theta; S1, primary somatosensory cortex; S2, secondary somatosensory cortex; M1, primary motor cortex. Significant correlations (α = 0.05) are shown in bold.

## Data Availability

The data sets analyzed during the current study are available from the corresponding author on reasonable request. The custom code used for cluster analysis is publicly available on the TelkesLab GitHub repository: scs_preop_eeg_clustering.
