# Supplementary material for: EEG-based clustering shows distinct separation of chronic pain patients before spinal cord stimulation surgery
Source: Neuroimage. Author manuscript; Available in PMC 2026 Jun 18. (PMC13276974; doi:10.1016/j.neuroimage.2026.122002)
Supplement: 1 [file NIHMS2185408-supplement-1.docx]

**Supplementary Material**

| **Supplementary Table S1.** Individual-Level Demographic and Clinical Characteristics Across Clusters | | | | | | | | | | | | | |
| --- | --- | --- | --- | --- | --- | --- | --- | --- | --- | --- | --- | --- | --- |
| **Subject ID** | **Sex**  **(M/F)** | **Age** | **Diagnosis** | **Disease duration (years)** | **Baseline MME (mg)** | **Responder**  **(Yes/No)** | **NRS worst** | **NRS average** | **BDI** | **MPQ total** | **PCS total** | **ODI** | **Cluster** |
| P1 | M | 77 | PSPS type 2 | 12 | 5 | Yes | 6 | 6 | 11 | 6 | 21 | 36 | 1 |
| P2 | M | 58 | PSPS type 2 | 12.3 | 22.5 | Yes | 8 | 7 | 0 | 6 | 7 | 18 | 1 |
| P3 | M | 53 | PSPS type 1 | 20 | 20 | Yes | 8 | 7 | 14 | 3 | 14 | 48.9 | 2 |
| P4 | F | 57 | PSPS type 2 | 4 | 4.5 | No | 8 | 5 | 19 | 5 | 10 | 35.6 | 2 |
| P5 | M | 68 | PSPS type 1 | 40 | 7.5 | No | 8 | 6 | 27 | 7 | 39 | 70 | 2 |
| P6 | F | 66 | PSPS type 1 | 13 | 82.5 | Yes | 9 | 8 | 17 | 9 | 12 | 62 | 2 |
| P7 | F | 79 | PSPS type 2 | 15 | 30 | Yes | 10 | 8 | 26 | 5 | 20 | 52 | 2 |
| P8 | F | 65 | PSPS type 1 | 16.9 | 30 | No | 9 | 7.5 | 11 | 10 | 38 | 57.8 | 2 |
| P9 | M | 40 | PSPS type 1 | 21 | 0 | Yes | 10 | 9 | 44 | 4 | 45 | 70 | 3 |
| P10 | F | 70 | PSPS type 1 | 17.5 | 20 | No | 8 | 2 | 5 | 3 | 6 | 37.8 | 3 |
| P11 | F | 69 | PSPS type 1 | 19.6 | 0 | Yes | 10 | 8 | 7 | 5 | 0 | 28 | 3 |
| P12 | F | 59 | PSPS type 1 | 13 | 60 | Yes | 8 | 9 | 10 | 3 | 21 | 64 | 3 |
| P13 | F | 75 | PSPS type 1 | 16.9 | 52.5 | Yes | 9 | 8 | 31 | 8 | 43 | 72 | 3 |
| P14 | F | 83 | PSPS type 2 | 24 | 0 | Yes | 8 | 7 | 10 | 11 | 26 | 34 | 3 |
| P15 | F | 47 | PSPS type 1 | 5 | 40 | No | 7.5 | 7.5 | 6 | 6 | 30 | 52 | 3 |
| P16 | F | 66 | PSPS type 2 | 6 | 0 | Yes | 8 | 4.5 | 16 | 1 | 27 | 45 | 3 |
| *PSPS, Persistent Spinal Pain Syndrome; MME, morphine milligram equivalents. NRS, Numerical Pain Rating Scale; ODI, Oswestry Disability Index; PCS, Pain Catastrophizing Scale; MPQ, McGill Pain Questionnaire; BDI, Beck’s Depression Inventory.* | | | | | | | | | | | | | |
